# Attitudes of Iranian Psychiatrists to Psychosomatic Medicine: A Qualitative Content Analysis 

**Published:** 2018-10

**Authors:** Imanollah Bigdeli, Sahar Mohabbat-Bahar, Amirreza Boroumand, Ali Talaei, Farzad Goli

**Affiliations:** 1 Department of Psychology, Faculty of Education and Psychology, Ferdowsi University of Mashhad, Mashhad, Khorasan Razzavi, Iran.; 2 Department of Psychosomatic, Freiburg University, Freiburg, Germany.; 3 Psychiatry and Behavioral Sciences Research Center, Mashhad University of Medical Sciences, Mashhad, Khorasan Razzavi, Iran.; 4 Energy Medicine University, California, USA.

**Keywords:** *Attitude*, *Integrative Medicine*, *Psychiatry*, *Psychosomatic Medicine*, *Qualitative Research*

## Abstract

**Objective:** Psychosomatic attitudes may be rooted and grounded in the particular culture of the scientific community in each country. We conducted a qualitative research to understand the exclusive psychosomatic attitudes and psychosomatic medicine status of Iranian Psychiatrists.

**Method** **:** This research was conducted using a qualitative content analysis based on Graneheim and Lundmanand method. All psychiatrists of Avicenna hospital, which is a teaching hospital of Mashhad University of Medical Sciences, were the target population of this study. Among them, 9 psychiatrists were selected by a non-random purposeful sampling method, and semi-structured interviews were used to collect data.

**Results: **Results indicated that most psychiatrists do not have a proper understanding of the term “psychosomatic medicine”, but they acknowledged the importance of an integrative approach in medicine. Biopsychosocial model, as a unified and integrated concept, can encompass all emerged categories and refers to the overall pattern in psychiatrists' attitude.

**Conclusion: **Despite acknowledging the importance of a comprehensive approach to medicine and profound theoretical knowledge of psychiatrists, the practical application of biological, psychological, and social dimensions has not been considered equally. Thus, the scientific practical stand of this comprehensive approach requires more serious consideration by the medical community.

Mind-body interventions are defined as methods that focus on the interactions among the brain, mind, body and behavior. This domain tends to use the mind to make changes in physical function and general health condition (1). Mind-body medicine is based on physical, psychological, social, and spiritual aspects (2). It is also an effective and relatively practical model that can be applied to clinical practice through 5 levels: physical, emotional, cognitive, behavioral, and spiritual. Each pole requires research, assessment, training, awareness, unique techniques, and treatments (3). Mind-body medicine focuses on the mental health needs of people who suffer from a general medical condition (4). Patients will experience specific physiological benefits when they practice such techniques as relaxation, meditation, imagery, autogenic training, hypnosis, and self-expression. The beneficial consequences are reduction in stress and pain, increase in sleep quality, mood improvement, decrease in stress hormone levels, and immune system enhancement. When patients feel the benefits of the mentioned technique, they feel in control and can overcome frustration and hopelessness (5). There is considerable evidence that a series of mind-body therapies can be used as an effective and complementary approach in rehabilitation and treatment of various diseases (6-10). George Engel in 1977 emphasized on an integrated approach to human behavior and disease.

Biopsychosocial model, which he described, was a reaction to limited consideration of medical and other basic science advances in biomedical model.

Biological, psychological, and social factors can influence the prevention, causes, experiences, management, and treatment of diseases. These factors constantly interact with each other and constitute a special situation, which is known as a disease (11). The biopsychosocial model is considered a philosophy and also a clinical care guide. From a philosophical viewpoint, this model is a way to understand how illness is affected by multiple levels of an organization, from a molecular to a social level. Therefore, each level must be considered as an essential factor in accurate diagnoses, health, and human care. This comprehensive approach is also necessary to understand the subjective experience of patient's disease (12). Sulmasy (13) aimed at investigating the role of biopsychosocial models at the patients' end of life and emphasized that, in addition to the biological aspects, the illness affects all aspects of a person's existence, such as psychological, social, and even spiritual. He also concluded that a comprehensive framework should be considered by professionals to develop a holistic health care. 

Lumley et al. (14) demonstrated how the pain may be modulated by emotional states. They concluded that pain catastrophizing, pain-related fear and anxiety, social rejection, insecure attachment, and high arousal negative emotions are related to greater pain and maladaptive adjustment. These emotional factors occur not only in response to pain, but also trigger, maintain, or exacerbate pain. Neuroscience research supports the assumption that pain may be influenced by negative emotional factors, particularly via the medial pain system and its projections to the anterior cingulate cortex, amygdala, and medial prefrontal cortex (14). Thus, health care providers should pay attention to pain modulators and try to eliminate destructive emotions in their clinical interventions.

Psychosomatic medicine, a specialty of medicine, is a comprehensive interdisciplinary framework for assessment of all psychosocial factors affecting individual vulnerability, course, and outcome of any type of disease. It is also known by comprehensive medical care and integration of various psychological treatments in the prevention, treatment, and rehabilitation of medical conditions (15). Psychosomatic care, which can be integrated into daily medical practice, has considerable outcomes, including patient satisfaction, amount of drug prescription, compliance with the treatment regimen, the therapeutic outcome, and professional satisfaction (16). Despite the importance of the psychosomatic approach in the treatment of physical and psychological illnesses, this powerful phenomenon has been less considered, and we could not find any studies in Iran to investigate psychosomatic attitudes among mental health providers.

Psychosomatic attitudes may be rooted and grounded in the culture of the scientific community in each country. The present study aimed at filling this gap by a qualitative study. We conducted a qualitative study to understand the exclusive psychosomatic attitudes and psychosomatic medicine status among Iranian psychiatrists. Furthermore, this study has been performed to pursue 2 objectives: (1) obtaining a deep understanding of the role of psychosomatic medicine, and (2) analyzing the psychosomatic medicine elements by a qualitative method.

## Materials and Methods

***Study Design***

This study was conducted using a qualitative content analysis based on Graneheim and Lundman (17) method for analyzing narrative data. They introduced a systematic and objective method that is applicable in health research. 

***Participants***

All psychiatrists of Avicenna hospital, which is a teaching hospital of Mashhad University of Medical Sciences, were the target population of this study. Among them, 9 psychiatrists were interviewed until data saturation. They were selected by a non-random purposeful sampling method. Inclusion criteria for participants were as follows: (1) at least 5 years of experience in a psychiatric hospital and (2) tendency to participate in the research.

***Procedures***

Semi-structured interviews were used to collect data. About 20 to 40 minutes were allocated to each participant and this process continued until data saturation. Data were collected by taking notes and audio recording. Interviews were performed face to face after obtaining informed consent. Interviews were done by a trained interviewer whose work was supervised by a specialist educated in a psychosomatic medicine fellowship. Participants were assured of non-disclosure of their identity. They were aware of the confidentiality of the data and the right to withdraw from the study. Interviews covered personal information and main questions. In some cases, the interviews were held in 2 sessions to clarify and complete the received information. The questions were designed around the 7 following domains: assessment and diagnosis, etiology, treatment plan, relapse, therapeutic alliance, patients' resources, and psychosomatic medicine. 

***Analysis***

According to the principles of Graneheim and Lundmanand content analysis method, we tried to avoid preset classification. The interviews were read several times to obtain a sense of the whole. Then, the text about the psychiatrists’ experiences of the 7 mentioned domains was brought together into 1 text, which constituted the unit of analysis. Meaning units, condensed meaning units, and codes are presented in Table 1. The condensed meaning units were abstracted by the meaning units and labeled as related codes. Various codes were based on differences and similarities and were sorted into 7 subcategories and 3 categories. Finally, the latent content was abstracted and formulated into a theme. Examples of codes, subcategories, categories, and a theme are demonstrated in Table 2. 

To ensure trustworthiness of the results, Lincoln and Guba (18) criteria were used to ensure the trustworthiness of the qualitative research. Lincoln and Guba (18) criteria introduce the 4 following criteria: (1) credibility, (2) transferability, (3) dependability, (4) confirmability. To meet the mentioned criteria, prolonged contacts were made by the participants and continuous observations were done in the research environment. Data were checked by colleagues, and separate coding was done by 2 members of the research team. Also, concepts were reviewed and approved by professors who were not involved in this study. Finally, in addition to the limited review of the literature, we tried to avoid subjective assumption interference in all processes of collecting and coding the interviews. 

## Results

Table 1 demonstrates the meaning units, condensed meaning units, and codes. Table 2 displays the theme, categories, subcategories, and related codes.

Participants' responses were analyzed in 7 areas (Table 1). In the assessment and diagnosis domain, differential diagnosis of physical illness, differential diagnosis of mental disorder, and comprehensive assessment of psychosocial status was shown and named with respect to the condensed analysis units. In etiology, 2 codes were extracted from psychiatrists' responses, which were biogenetic causes and diathese stress model. According to the participants' responses to questions that were related to treatment plan, psychotherapy and medication were identified. We classified relapse factors into 3 codes: (1) adherence, (2) biological relapse and social problems, and (3) interpersonal conflicts. Psychiatrists' experiences of therapeutic alliance indicated that they confirmed potential components that must be present in the doctor-patient relationship to achieve therapeutic purposes. Conformance and trust were influential components which were entered the communication by patients. Authority and sympathy were also physician resources that were thought to be existed. In addition to personal resources, psychiatrists also pointed to other economic and social factors in patient resources domain. Thus, 6 codes were derived from analysis units: (1) former coping strategies, (2) positive attitude, (3) insight, (4) social support, (4) economic resources, and (6) appropriate interpersonal relationship. Finally, participants were asked to provide a definition of psychosomatic medicine and establish the position of this field in practice. Psychosomatic medicine and psychosomatic disorders were classified as related codes. The connection between psychological factors and physical discomfort was noted in psychosomatic medicine. Also, participants acknowledged the role of psychosomatic medicine in all areas of assessment, prevention, and treatment. However, some psychiatrists thought that the psychosomatic medicine is equal to psychosomatic disorders, which nowadays have a high prevalence.

After comparing the extracted codes based on their similarities and differences, they were divided into 7 subcategories (biological assessment, biological intervention, patients' psychological resources, therapeutic alliance, interpersonal factors, sources in secondary priority, environmental pressures, and interpersonal factors) and 3 main categories (biogenetic factors, psychological factors, and socioeconomic factors). After consulting other colleagues, we tried to elicit the latent content as the main theme. Biopsychosocial model, as a unified and integrated concept, can encompass all categories and refers to the overall pattern in psychiatrists' attitudes (Table 2).

## Discussion

The results of this study revealed that psychiatrists have a biopsychosocial approach as a holistic perspective for the evaluation, diagnosis, etiology, and treatment of mental disorders. Psychiatrists emphasized the differential diagnosis in the first assessment session because most patients were concerned about the multiple physical and psychiatric symptoms. Participants also acknowledged that in addition to physical and psychological symptoms based on the diagnostic interview, if they had enough time, they would have performed a comprehensive assessment of patients' psychosocial status. As emphasized in DSM-5, ruling out a general medical diagnosis is the most important and difficult distinction in psychiatry because most patients experience psychiatric symptoms caused by a general medical problem and, in contrast, many psychiatric symptoms may arise from an underlying medical problem (19). On the other hand, health theorists have formulated an integrated approach for diagnosis, which has not gained a definite position in medicine. This approach is based on the phenomenological thought and mentalization as psychological concepts, which are related to understanding others. Understanding the patients' experiences and feelings are the essential components of mentalization process. Physicians try to keep them close to patients' subjectivity to provide a comprehensive understanding of the patient's status. Diagnostic classification of mental and physical symptoms is less considered in this approach (20, 21). 

The findings of this study also indicated that psychiatrists do not apply this approach in practice.

The emphasis on predetermined classification of mental disorders, differential diagnosis, and limited time allocated for assessment are evidences of the absence of this comprehensive approach. Consistent with our study, Davidsen & Fosgerau (22) aimed at investigating psychiatrists’ responses to emotions of depressed patients and they found that psychiatrists attempt to clarify symptoms by rational argumentation or by offering an interpretation of the emotions from their own perspectives, not from that of patients.

Psychiatrists tend to consider biogenetic factors and diathesis stress model as the main causes of mental disorders. There were also similar attitudes in treatment and relapse. Other studies have also supported diathesis stress model, including cognitive, emotional, and behavioral components as the predisposing factors in the etiology and relapses of various physical and mental disorders, such as chronic pain, depression, suicide, and panic (23-26). Participants assigned medication as the first and best therapeutic option and in some cases, psychotherapy in the second priority. They also mentioned non-adherence, the recurrent nature of psychiatric disorders, social problems, and interpersonal conflicts as the relapse causes. Psychiatrists assumed an influential role for biological causes and brain interactions, thus the important role of medication has been acknowledged in medicine. Medicine is prescribed by psychiatrists to eliminate and relieve psychological syndromes immediately. Also, referring to a clinical psychologist was noted by some participants as a complementary treatment. Writing a prescription for patients does not take place in a vacuum and the psychiatrist is not a medicine-dispensing machine; however, the role of the psychiatrist often includes providing psychopharmacologic evaluation, and psychotherapy is performed by a nonmedical allied mental health professional in many clinical settings (27). The results indicated that the combination of psychotherapy and medication work better than either treatment alone (28). 

Physician-patient relationship is the foundation of efficient medical care. Since the diagnostic information is obtained through interviews, if there is an optimal relationship treatment, patients may offer more detailed and comprehensive information, and thus therapeutic purposes will be achieved with precision. Compliance and lack of resistance to psychiatric treatments, trusting the psychiatrists’ knowledge and expertise, and physicians' empathic understanding and authority were the indisputable obligation of the therapeutic relationship, which were extracted through the interviews. Results of a meta-analysis about the effects of therapeutic alliance in patients' physical rehabilitation showed that the collaborative bond between the patient and therapist results in 4 therapeutic consequences: (1) adherence, (2) depression symptoms reduction, (3) treatment satisfaction, and (4) physical function improvement (29). The results have also revealed that compliance with medical instructions is strongly influenced by the physician-patient relationship (30). Empathy is also a therapeutic alliance component, which was defined as a predominately cognitive feature. Empathy refers to exclusively understanding patients' experiences, concerns, and views (31). Generally, the results indicated that empathic understanding and trust are important prerequisites for achieving optimal outcomes in the treatment of mental disorders and chronic diseases. Empathy leads to satisfaction and empowerment of patients and it may also reduce patients' anxiety and distress (32-35). One of the most important factors in a physician-patient relationship is patients' resources. In addition to therapeutic alliance, these resources also affect the treatment process and therapeutic purposes. Extraction of former coping strategies that the patient was using to manage past life crises, lack of resistance to the psychiatry, positive attitude, and insight are influencing resources, which may enhance the therapeutic goals. In addition to personal sources, participants considered the social and economic resources as facilitating and complementary factors. Fewer social problems and better insight can enrich therapeutic alliance (36). Consistent with our study, the results of other studies revealed that knowledge about psychiatric disorders, problem-focused coping strategies, and social support are patients' personal and social resources (37). Generally, function of a patient who has a psychiatric disorder may be affected by personal and social factors simultaneously, and thus paying attention to this point is of high importance in addressing integrated treatment (38). 

In addition to indirect investigation of psychosomatic medicine in the above-mentioned areas, psychiatrists' attitude to the psychosomatic medicine was also examined directly. The results showed that only 2 psychiatrists were familiar with the term “psychosomatic medicine” and most of the participants thought this term was equal to psychosomatic disorders. However, most psychiatrists do not have a proper understanding of this specific term, but are perfectly familiar with its more general model: the biopsychosocial approach. Psychosomatic medicine has a rigorous therapeutic role in the current medical research and practice owing to lifestyle changes, unexplained medical challenges, psychosocial needs of chronic patients, assessments beyond the pharmaceutical reductionism, and patients' active role in their own health (39). The absolute role of this approach was confirmed by participants with a similar biopsychosocial approach. Despite acknowledging the importance of a comprehensive approach to medicine and profound theoretical knowledge of the psychiatrists, the practical application of biological, psychological, and social dimensions has not been considered equally. In some cases, the biological aspect was highlighted and psychiatrists neglected the other 2 aspects. As Benning (40) concluded, despite the influence of this modern psychiatric model in both clinical medicine and educational systems, lack of its philosophical coherence, insensitivity to patients' subjectivity, and being unfaithful to the original frame of biopsychosocial theory that Engel claimed, made this model far from achieving its main goals.

**Table 1:** The Meaning Units, Condensed Meaning Units, and Codes Obtained from Qualitative Content Analysis

	**Meaning Units**	**Condensed Meaning Units**	**Codes**
Assessment and diagnosis	I emphasized the observable and considerable physical symptoms which may create suspicion that there is a hidden medical problem	Removing the possible diagnosis of physical diseases	The differential diagnosis of physical illness
	The patients' chief concern about the present annoying psychological symptoms is critical for accurate diagnosis Patient descriptions of psychological symptoms must be expanded	Focusing on the signs in the present moment	The differential diagnosis of mental disorder
	If I have extra time, I try to obtain information about interpersonal and social domains	Checking out the family conflicts and environmental stresses	Comprehensive assessment of psychosocial status
Etiology	There is a basic biological cause in all psychiatric disorders	Deterministic biological causes	Diatheses stress model
	Environmental stresses are triggers for those who have biological and genetic background	Stress is a trigger for those who are prone to mental disorder	Biogenetic causes
Treatment plan	Medication is our priority to relieve psychological suffering and distress immediately	Medications to relieve psychological symptoms	Medication
	Psychotherapy by a clinical psychologist is also recommended for some patients who have interpersonal conflicts and environmental stresses	Psychotherapy when there are interpersonal problems	Psychotherapy
Relapse	Cessation of drug use, lack of sufficient drug dose, and lack of follow up treatment are important reasons in relapse	Failure to follow the correct medication instructions	Adherence
	The recurrent nature of psychiatric disorders causes relapse, even without a trigger Sometimes relapse is out of the physician and patient’s control, such as seasonal disorders and hormonal changes Relapse may be due to a medical condition or drug abuse	The recurrent nature of mental disorder, basic biological causes, and recurrence because of other medical conditions	Biological relapse
	If problems remain unsolved, they could lead to relapse Interpersonal conflicts and emotional frustration, even after successful treatment, may play an important role in relapse Sometimes patients discontinue the drug because of resistance against family members, and this could lead to relapse	Problems that arise from the social environment and patient's interpersonal conflicts with family members	Social problems and interpersonal conflicts
Therapeutic alliance	An essential prerequisite for instantaneous recovery is acceptance and conformity Adherence to medication and regular visitation is required for recovery	Therapy session continuation and adherence to medication prescriptions	Conformance
	Ethical standards, such as spending adequate time for the patient, facilitate the treatment process If there is an effective physician-patient communication, patients will not interrupt the treatment process, even in case of adverse physical side effects The patient must believe that the physician has adequate knowledge and expertise for effective treatment	Trusting the treatment process and the physician’s prescriptions will lead to recovery	Trust
	Patients believe that the physician understands their distress If there is a therapeutic alliance, we will be able to collect more comprehensive information for accurate diagnosis	Patients' perception of empathy	Sympathy
	We must act strictly in emergency cases and when the patient does not have insight The patients consider the physician as a source of authority We must have a paternalistic approach when hospitalization is indispensable	Adherence to undisputed medical prescriptions	Authority
Patients resources	By a solution-focused approach, we will be aware of patients' capabilities in managing previous crises through interviews	Extraction of successful strategies in dealing with previous problems	Former coping strategies
	It is essential that patients enter the treatment process with positive attitudes about acquiring mental health once again Personality disorders are an obstacle to patients' acceptance	Positive attitude towards psychiatric treatment	Positive attitude
	The patient must have adequate intelligence Patients should have Knowledge about risk factors	Patients awareness about their psychological status	Insight
	A non-stigmatized community approach to mental disorder The possibility of joining self-help groups for patients	Available supportive resources in community	Social support
	Understanding and accompanying patients during treatment and convalescence by family members	Having an appropriate relationship with family members and friends	Appropriate interpersonal relationship
	Patients who have a job and sufficient income have no economic problems to continue the treatment courses	Sufficient level of economic prosperity and welfare	Economic resources
Psychosomatic medicine	Nowadays, due to mechanization of life, the prevalence of patients who refer to neurologists and psychiatrists because of physical pain is increasing This field is practically limited to physical disorders combined with the influencing role of psychological factors This field is a connection between medicine and psychiatry	Physical disorders which have been influenced by psychological factors	psychosomatic disorders
	Consultation liaison, a branch of psychosomatic medicine, highlights the demand of addressing the psychological issues Biological, psychological, and social factors have an interaction in assessment, prevention, and treatment	The importance of psychosocial needs of patients with a medical condition	Psychosomatic medicine

**Table 2:** Theme, Categories, Subcategories, and Related Codes Obtained from Qualitative Content Analysis

**Theme**	**Biopsychosocial model**						
**Category**	**Biogenetic factors**		**Psychological factors**			**Socioeconomic factors**	
**Subcategory**	**Biological assessment**	**Biological ** **intervention**	**Patients' ** **Psychological ** **resources**	**Therapeutic ** **alliance**	**Sources in ** **secondary ** **priority**	**Environmental ** **pressures**	**Interpersonal ** **factors**
**Codes**	The differential diagnosis of physical illness The differential diagnosis of mental disorder Biogenetic causes Diatheses stress model Biological relapse Psychosomatic disorders	Medication	Former coping strategies positive attitude Insight Adherence	Conformance Trust Authority Sympathy psychosomatic medicine	Comprehensive assessment of psycho-social status Psychotherapy	Economic resources	Social support Appropriate interpersonal relations

## Limitation

This study has some limitations which must be mentioned. The sample size was small and the psychiatrists were selected from only one hospital, and thus bias might have occurred in the data collection process.

## Conclusion

We attempted to create a deep understanding of psychiatrists' attitudes towards psychosomatic medicine and identify its elements. This comprehensive framework can help address the psychological needs of the patients with medical problems and it can also improve the quality of comprehensive education in psychiatry. Moreover, it can promote biopsychosocial research in integrative health care. 

Despite the unfamiliarity with the term “psychosomatic medicine”, psychiatrists acknowledged the conclusive stand of biopsychosocial approaches in medicine. All the psychiatrists had a firm conviction about biological, psychological, social, and even spiritual dimension theoretically. However, the qualitative content analysis of the interviews indicated prioritizing biogenetic factors compared to other factors. In practice, allocating just a few minutes to achieve a diagnosis in the first assessment session may contradict with a holistic biopsychosocial approach. Emphasizing the visible biological signs, categorizing patients’ symptoms, and medication as the first choice of therapy are practical weaknesses of this approach. Thus, the practical scientific stand of this comprehensive approach requires more serious consideration by the medical community.
